# Failed noninvasive positive-pressure ventilation is associated with an increased risk of intubation-related complications

**DOI:** 10.1186/s13613-015-0044-1

**Published:** 2015-03-06

**Authors:** Jarrod M Mosier, John C Sakles, Sage P Whitmore, Cameron D Hypes, Danielle K Hallett, Katharine E Hawbaker, Linda S Snyder, John W Bloom

**Affiliations:** Section of Pulmonary, Critical Care, Allergy and Sleep, Department of Medicine, University of Arizona, 1501 N Campbell Ave., Tucson, AZ 85721 USA; Department of Emergency Medicine, University of Arizona, 1609 N. Warren Ave., Tucson, AZ 85724 USA; Division of Emergency Critical Care, Department of Emergency Medicine, University of Michigan Health System, 1500 E Medical Center Drive, Ann Arbor, MI 48109 USA; University of Arizona, 1609N Warren, FOB 122C, Tucson, AZ 85719 USA

**Keywords:** Intubation, Critical care, NIPPV, Noninvasive positive pressure, Airway management, Desaturation, Hypotension, Aspiration, Delayed intubation

## Abstract

**Background:**

Noninvasive positive-pressure ventilation (NIPPV) use has increased in the treatment of patients with respiratory failure. However, despite decreasing the need for intubation in some patients, there are no data regarding the risk of intubation-related complications associated with delayed intubation in adult patients who fail NIPPV. The objective of this study is to evaluate the odds of a composite complication of intubation following failed NIPPV compared to patients intubated primarily in the medical intensive care unit (ICU).

**Methods:**

This is a single-center retrospective cohort study of 235 patients intubated between 1 January 2012 and 30 June 2013 in a medical ICU of a university medical center. A total of 125 patients were intubated after failing NIPPV, 110 patients were intubated without a trial of NIPPV. Intubation-related data were collected prospectively through a continuous quality improvement (CQI) program and retrospectively extracted from the medical record on all patients intubated on the medical ICU. A propensity adjustment for the factors expected to affect the decision to initially use NIPPV was used, and the adjusted multivariate regression analysis was performed to evaluate the odds of a composite complication (desaturation, hypotension, or aspiration) with intubation following failed NIPPV versus primary intubation.

**Results:**

A propensity-adjusted multivariate regression analysis revealed that the odds of a composite complication of intubation in patients who fail NIPPV was 2.20 (CI 1.14 to 4.25), when corrected for the presence of pneumonia or acute respiratory distress syndrome (ARDS), and adjusted for factors known to increase complications of intubation (total attempts and operator experience). When a composite complication occurred, the unadjusted odds of death in the ICU were 1.79 (95% CI 1.03 to 3.12).

**Conclusions:**

After controlling for potential confounders, this propensity-adjusted analysis demonstrates an increased odds of a composite complication with intubation following failed NIPPV. Further, the presence of a composite complication during intubation is associated with an increased odds of death in the ICU.

## Background

The use of noninvasive positive-pressure ventilation (NIPPV) for acute respiratory failure has increased over the last two decades [1,2] and has shown a benefit in the treatment of acute respiratory failure due to chronic obstructive pulmonary disease (COPD), congestive heart failure (CHF), and in immunocompromised patients [3-12]. Data for the use of NIPPV for other causes of acute respiratory failure such as asthma, pneumonia, or acute respiratory distress syndrome (ARDS) are scant [13,14]. Despite the lack of evidence, NIPPV is still often used in these conditions [2], while outcomes data such as mortality, rate of intubation, and other complications following the use of NIPPV for the treatment of respiratory failure are concerning [2,14-23].

While NIPPV use has been shown to decrease the need for intubation in patients with respiratory failure regardless of etiology, there are no data regarding the risk of intubation-related complications associated with delayed intubation in adult patients who fail NIPPV. Several studies have demonstrated an increase in mortality with delayed intubation after failed NIPPV in acute respiratory failure and after failed extubation [1,15,17,18]. Given the limited physiologic reserve and high risk of hypoxemia, hemodynamic deterioration, and even cardiac arrest with intubation in critically ill patients [24-30], complications experienced with delayed intubation in these patients may partially explain the mechanism for the increased mortality seen with failed NIPPV requiring intubation. While hemodynamic deterioration, hypoxemia, and aspiration are risks of intubation in all critically ill patients, this study seeks to evaluate if intubation after failed NIPPV increases this risk when compared to patients intubated in the medical intensive care unit (ICU) without a trial of NIPPV. Some of the results of this study have previously been presented in abstract form [31].

## Methods

### Study design

This is an observational study comparing patients intubated after failed NIPPV versus those patients intubated without undergoing a trial of NIPPV. The failed NIPPV group includes all patients intubated after initially undergoing NIPPV prior to intubation identified in our continuous quality improvement (CQI) database from 1 January 2012 to 30 June 2013. The primary intubation group was selected from the CQI database for comparison. This project was reviewed and approved by the university’s institutional review board.

### Setting and population

This study was conducted at a major academic referral center with a 20-plus bed medical ICU, which is staffed by two teaching teams. This ICU service is affiliated with ACGME-accredited 3-year pulmonary/critical care medicine (Pulm/CCM) and 2-year critical care medicine (CCM) fellowship programs with a total of 16 fellows. Each teaching team is staffed with an attending (Pulm/CCM or CCM), a fellow (postgraduate year (PGY) 4 to 6), and resident (internal medicine PGY 1 to 3 and emergency medicine PGY 2) physicians. Occasionally, fellows from anesthesiology or surgical critical care fellowships rotate through the medical ICU service. All intubations are performed under attending supervision. For the duration of the study period, direct laryngoscopes were available in all sizes of Macintosh and Miller blades. Video laryngoscopes were available throughout the entire study period and are the preferred device used in the medical ICU. The following video laryngoscopes were available during the study period: GlideScope (GVL) (Verathon, Bothell, WA, USA) with both reusable and disposable blade configurations in sizes 3 and 4, and the C-MAC (Karl Storz, Tuttlingen, Germany) with Macintosh-type blade sizes 3 and 4. In addition to direct and video laryngoscopes, flexible fiber-optic bronchoscopes are available and occasionally chosen as the initial device for suspected anatomically difficult airways.

### Selection of participants

All patients intubated on the medical ICU service are recorded in a CQI database. Following each intubation, the operator completes a data collection form, which includes information regarding patient and operator demographics, circumstances of the intubation including presence and duration of NIPPV therapy prior to intubation, indication for intubation, devices and pharmacologic agents used for intubation, presence of certain difficult airway characteristics (DACs), pre-oxygenation methods, the number of attempts at intubation, the outcome of each attempt, and complications. All patients intubated after failing NIPPV for acute respiratory failure were included in the study and compared to patients intubated without a trial of NIPPV. Since cardiac arrest patients were not eligible for consideration for NIPPV, they were excluded from this analysis.

### Methods of measurement

The following information was extracted from the patients’ medical record: pre- and post-intubation vital signs, pre- and post-NIPPV and intubation blood gas data, severity of illness (simplified acute physiology score (SAPS II) and acute physiology and chronic health evaluation (APACHE II and IV)) at the time of intubation, and outcomes data including ventilator days and ICU mortality. Patient and operator demographics as well as the circumstances regarding the intubation were obtained from the CQI collection form including operator postgraduate year, method of intubation, difficult airway characteristics (DACs), and device(s) used, as well as the attempts and outcome of each attempt. Methods of intubation included rapid sequence intubation (RSI) in which a paralytic agent was used, oral intubation in which a sedative agent only was used (SED), and oral intubation in which no medications were used (OTI).

We utilized a list of DACs that were chosen because they are feasible for the operator to determine prior to intubation in an emergent setting by simple examination of the patient. These include both anatomic and physiologic DACs. The anatomic DACs that make either visualization of the glottic inlet or placement of a tracheal tube difficult included the presence of blood, vomit, or secretions in the airway, cervical immobility (intrinsic or due to a cervical collar), obesity, a large tongue, a short neck, a small mandible, facial or neck trauma, airway edema, and limited mouth opening. Physiologic DACs included hemodynamic instability and hypoxemia, which may make the process of intubation more challenging and are likely to influence the intubation plan.

We defined NIPPV failure as the need for intubation, despite NIPPV support, whether due to refractory hypoxemia, hypoventilation, work of breathing, decline in mental status, failure to tolerate the device, or a change in treatment plan at the provider’s discretion. Complications evaluated include hypotension, desaturation, esophageal intubation, aspiration, airway trauma, and ‘others.’ For this analysis, hypotension, desaturation, and aspiration were considered to be the important complications of interest in that they may plausibly be affected by delayed intubation after a trial of NIPPV. The other monitored complications were felt to be equally likely to occur between the two groups as they are more related to the procedure and independent of the physiology. We defined hypotension as any drop in blood pressure requiring intervention such as fluid resuscitation or initiation or titration of vasopressors that occurred during or within 5 min of the intubation. Desaturation was defined as a decline in oxygen saturation >10% from the baseline during the intubation procedure. Aspiration included any witnessed aspiration of gastric contents during the intubation attempt. For this analysis, these three complications were combined into a composite complication to represent the risk of delayed intubation after a failed NIPPV trial.

The data collection forms were reviewed by the primary author for completion following the procedure. If the forms had any missing data that could not be obtained from the medical record, they were returned to the operator for completion. If information on the form contained inconsistencies, the operator was interviewed by the primary author for clarification.

The data were then entered into the electronic database (Excel for Macintosh 2011 (Microsoft, Redmond, WA, USA)) and transferred to Stata for analysis (Stata version 12; StataCorp, College Station, TX, USA).

### Outcome measures

The primary outcome measured was the incidence of a composite complication (desaturation, hypotension, or aspiration) during intubation. The secondary outcome measured was the odds of death in the ICU following the occurrence of a composite complication during intubation.

### Primary data analysis

Summary statistics were generated for patient, intubation, and operator characteristics using Fisher’s exact test for categorical variables, the Kruskal-Wallis test, and Student’s *T*-test where appropriate. A propensity score for receiving NIPPV for respiratory failure was generated from prespecified variables expected to affect the decision to use NIPPV and included respiratory failure after failed extubation within the last 72 h, blood present in the airway, vomit present in the airway, obesity, hemodynamic instability, hypoxemia, and SAPS II. A propensity-adjusted multivariate logistic regression model was chosen for this analysis because it was thought that certain variables might affect both the clinician’s decision to use NIPPV versus invasive mechanical ventilation and simultaneously affect the likelihood of a composite complication, thus, introducing confounding. Propensity adjustment seeks to adjust the model to eliminate this type of confounding on the basis of treatment assignment. The analysis is performed by first generating a propensity score estimating the probability of treatment assignment then performing a logistic regression adjusted for the propensity score. This has the effect of reducing the influence of nonrandomized treatment selection and, assuring that conditional on the propensity score, the distribution of measured baseline covariates will be similar between the two treatment groups [32].

A propensity-adjusted multivariate logistic regression analysis was then performed modeling the odds of a composite complication [32]. The predictor variable of interest was failed NIPPV. Other variables that were likely to confound the occurrence of a composite complication were added to the model in a stepwise fashion and included the presence of pneumonia or ARDS, total attempts at intubation, device used for intubation, reason for intubation, and postgraduate year of the operator performing the intubation. Variables were removed if the odds ratio of the predictor variable did not change by more than 0.1. A Hosmer-Lemeshow test was performed on the propensity-adjusted model for goodness of fit. An unadjusted odds ratio was calculated for the secondary outcome of interest (risk of a death in the ICU) with the occurrence of a composite complication during intubation. Descriptive statistics were performed on measured variables with reported means, standard deviations, medians, and interquartile ranges (IQR) where appropriate.

## Results

Over the 18-month study period, a total of 287 patients were intubated. Five patients were excluded due to cardiac arrest, and 47 patients were excluded for incomplete data. Of the remaining patients, 125 patients were intubated after failed NIPPV and constituted the failed NIPPV cohort. Of these 125 patients, 28 patients were given a trial of NIPPV for respiratory failure after extubation within the preceding 72 h. The remaining 110 patients were intubated without a trial of NIPPV and were selected for comparison. Table 1 summarizes the baseline patient and operator demographics. There were no differences in age or gender between groups. There were few differences in difficult airway characteristics between the two groups. Fewer patients intubated after failed NIPPV had blood present in the airway (7/125, 5.6% vs. 25/110, 22.7%; *p* < 0.001), airway edema (5/125, 4.0% vs. 15/110, 13.6%; *p* = 0.01), or hypoxemia defined as oxygen saturation <88% (26/125, 20.8% vs. 43/110, 39.1%; *p* = 0.003) compared to patients intubated primarily. However, more patients in the failed NIPPV group had a short neck (47/125, 37.6% vs. 22/110, 20.0%; *p* = 0.004) compared to patients intubated primarily (Table 1).

**Table 1:** **Patient demographics**

**Characteristics**	**Primary intubation (** ***n*** **= 110)**	**Failed NIPPV (** ***n*** **= 125)**	***p*** **value**
Mean age, years	58.1 (IQR 50 to 67)	61.2 (IQR 54 to 73)	0.13
Gender			
Male	57% (63)	61% (76)	0.60
DACs			
Total DACs (median)	2 (IQR 1 to 3)	2 (IQR 1 to 3)	0.83
None	21.3% (23)	24% (30)	0.64
Cervical immobilization	3.6% (4)	4.8% (6)	0.75
Blood in airway	22.7% (25)	5.6% (7)	<0.001
Vomit in airway	4.6% (5)	4.8% (6)	1.00
Facial/neck trauma	0.9% (1)	0.8% (1)	1.00
Obesity	27.3% (30)	37.6% (47)	0.10
Short neck	20.0 % (22)	37.6% (47)	0.004
Large tongue	13.6% (15)	20.0% (25)	0.23
Airway edema	13.6% (15)	4.0% (5)	0.01
Small mandible	13.6% (15)	18.4% (23)	0.38
Hypoxemia	39.1% (43)	20.8% (26)	0.003
Hemodynamic instability	24.6% (27)	20% (25)	0.43
Limited mouth opening	8.2% (9)	12% (15)	0.39
Secretions	9.1% (10)	12.8% (16)	0.41
Illness severity			
Mean (median, IQR)			
APACHE II	19.4 (18, IQR 14 to 24)	15.2 (14, IQR 11 to 18)	<0.001
Mortality mean/median	35.9% (29.1%)	24.3% (19%)	<0.001
SAPS II	46.9 (46.5, IQR 37 to 57)	40.1 (38, IQR 31 to 49)	
Mortality mean/median	41.6% (38%)	29.4% (21%)	<0.001
APACHE IV	85 (85.5, IQR 64 to 102)	73 (69, IQR 57 to 90)	
Mortality mean/median	37.4% (36.5%)	24.6% (20%)	
Hypoxemic respiratory failure	45.5% (50)	64%(80)	0.006
Pneumonia or ARDS	31% (34)	49% (61)	0.008

NIPPV, noninvasive positive-pressure ventilation; IQR, interquartile range; DACs, difficult airway characteristics; SAPS, simplified acute physiology score; APACHE, acute physiology and chronic health evaluation; ARDS, acute respiratory distress syndrome.

Patients that failed NIPPV had significantly lower severity of illness scores (APACHE II, APACHE IV, and SAPS II) than the group that was primarily intubated (*p* < 0.001, Table 1). The SAPS II score demonstrated the largest difference between the groups with the median score and mean predicted mortality being 38% and 29.4% in the failed NIPPV group versus 46.5% and 41.6% in the primarily intubated group (*p* < 0.001). Of patients intubated after failed NIPPV, 64% (80/125) were intubated for hypoxemic respiratory failure compared to 45.5% (50/110) of patients intubated primarily, *p* = 0.006. Nearly half of patients intubated after failed NIPPV (49% (61/125)) were intubated for either pneumonia or ARDS, whereas only 31% (34/110) of patients intubated primarily had pneumonia or ARDS, *p* = 0.008 (Table 1).

For the patients treated with NIPPV, the location of NIPPV initiation was the emergency department in 11.6%, the ICU in 77.7%, and the general medical ward in 10.7%. The most common reason for initiation of NIPPV was hypoxemia (55.9%), followed by hypercapnea (25.4%), and increased work of breathing (8.5%) (Table 2). The reason for intubation as selected on the data form was different between the two groups (*p* < 0.001). Most notably, 91.2% of the failed NIPPV group were intubated for respiratory failure versus 62.7% of the primarily intubated group. The primary intubation group was intubated more often than the failed NIPPV group for airway protection, hemodynamic instability, and severe acidosis (26.4% vs. 6.4%, 5.5% vs. 0.8%, and 3.6% vs. 0.8%, respectively) (Table 2). There were no significant differences in the method of intubation, number of intubation attempts, or the PGY level of the operator between the two groups (Table 2).

**Table 2:** **NIPPV and intubation demographics**

**Characteristics**	**Primary intubation (** ***n*** **= 110)**	**Failed NIPPV (** ***n*** **= 125)**	***p*** **value**
Reason for NIPPV			
Hypoxemia	-	55.9% (66)	-
Hypercapnea	-	25.4% (30)	-
Work of breathing	-	8.5% (10)	-
Other	-	10.2% (12)	-
Duration of NIPPV	-		-
Mean/median (h)	-	12.3/5.8	-
Reason for intubation^a^			<0.001
Airway protection	26.4% (29)	6.4% (8)	
Respiratory failure	62.7% (69)	91.2% (114)	
Patient control	1.8% (2)	0.8% (1)	
Hemodynamic instability	5.5% (6)	0.8% (1)	
Severe metabolic acidosis	3.6% (4)	0.8% (1)	
Method of intubation			0.25
RSI	73.6% (81)	69.6% (87)	
SED	25.5% (28)	30.4% (38)	
OTI	0.9% (1)	0.0% (0)	
Operator PGY level			0.27
1	12.8% (14)	7.2% (9)	
2	21.1% (23)	16.0% (20)	
3	14.7% (16)	12.8% (16)	
4	21.1% (23)	29.6% (37)	
5	16.5% (18)	24.8% (31)	
6	11.9% (13)	8.8% (11)	
Attending	1.8% (2)	0.8% (1)	

^a^Reason for intubation includes airway protection in which the patient is unable to protect the airway from aspiration of secretions; respiratory failure, which includes all etiology of respiratory failure; patient control which is defined as agitation, danger to self, or to facilitate evaluation/procedures; hemodynamic instability, which is defined as shock and severe metabolic acidosis.

NIPPV, noninvasive positive-pressure ventilation; RSI, rapid sequence intubation; SED, sedation-only intubation; OTI, orotracheal intubation without medication; PGY postgraduate year.

The first attempt success rate for primary intubation patients was slightly higher, but not statistically different (77.3% vs. 69.6%, *p* = 0.24) (Table 3). There were no significant differences in the mean number of ventilator days (primary intubation 7.85 IQR 3 to 9, failed NIPPV 9.0 IQR 3 to 10), number of ICU days (primary intubation 11.5 IQR 5 to 15, failed NIPPV 15.1 IQR 5 to 16), or mortality rate (primary intubation 35.5%, 95% CI 26.3 to 44.5; failed NIPPV 29.5%, 95% CI 21.5 to 37.7) (Table 3).

**Table 3:** **Intubation success, complications, and patient outcomes**

**Characteristic**	**Primary intubation %, (** ***n*** **= 110)**	**Failed NIPPV %, (** ***n*** **= 125)**	***p*** **value**
Intubation			
First attempt success	77.3% (85)	69.6% (87)	0.24
Total attempts (mean/median)	1.25 (1)	1.34 (1)	0.22
Outcomes (median (IQR))			
Ventilator days	5 (3 to 9)	4 (3 to 10)	0.52
ICU days	9 (5 to 15)	9 (5 to 16)	0.26
ICU mortality	35.5% (95% CI 26.4 to 44.5)	29.5% (95% CI 21.5 to 37.7)	0.40
Complications			
Hypotension	27.3% (30)	32% (40)	0.48
Desaturation	23.6% (26)	32% (40)	0.19
Aspiration	1.8% (2)	1.6% (2)	1.0
>1 complication	10% (11)	12.8% (16)	0.54

NIPPV, noninvasive positive-pressure ventilation; IQR, interquartile range; ICU, intensive care unit.

Table 3 demonstrates the complication rates between the two groups. There were no statistically significant differences in rates of hypotension (primary intubation 27.3%, 95% CI 19.2 to 36.6; failed NIPPV 32%, 95% CI 23.9 to 40.9) and desaturation (primary intubation 23.6%, 95% CI 16.1 to 32.7; failed NIPPV 32%, 95% CI 23.9 to 40.9). There were no differences in rates of aspiration (primary intubation 1.8%, 95% CI 0.2 to 6.4; failed NIPPV 1.6%, 95% CI 0.2 to 5.7) or the number of patients with more than one of the above complications (primary intubation 10%, 95% CI 5.1 to 17.2; failed NIPPV 12.8%, 95% CI 7.5 to 20) (Table 3). There was no difference in the percentage of intubations with a composite complication when patients failed NIPPV in <6 h (54%) versus >6 h (49%). The unadjusted odds of a composite complication (desaturation, hypotension, or aspiration) was 1.5 (95% CI 0.90 to 2.51). The propensity-adjusted multivariate regression analysis demonstrated that the odds of a composite complication in patients who fail NIPPV was 2.20 (CI 1.14 to 4.25) when corrected for the presence of pneumonia or ARDS and factors known to increase complications of intubation (total attempts and operator experience) (Table 4). The Hosmer-Lemeshow test demonstrates a good fit to the model (0.87). When a composite complication occurred, the unadjusted odds of death in the ICU were 1.79 (95% CI 1.03 to 3.12).

**Table 4:** **Odds of a composite complication with intubation**

	**Odds of a composite complication of intubation** ^**b**^			
	**Unadjusted (crude)**		**Adjusted** ^**a**^	
Variable	Odds ratio	95% CI	Odds ratio	95% CI
Failed NIPPV	1.50	0.90 to 2.51	2.20	1.14 to 4.25
Pneumonia or ARDS	2.09	1.23 to 3.55	1.67	0.93 to 2.99
Total attempts				
1	Reference		Reference	
2	2.17	1.16 to 4.03	2.35	1.19 to 4.64
3	3.34	0.63 to 17.72	3.41	0.60 to 19.37
Operator PGY				
1	Reference		Reference	
2	1.24	0.45 to 3.44	1.38	0.46 to 4.16
3	1.3	0.44 to 3.82	1.00	0.31 to 3.19
4	0.87	0.33 to 2.29	0.74	0.25 to 2.15
5	1.25	0.46 to 3.38	1.14	0.39 to 3.40
6	2.6	0.80 to 8.49	2.23	0.62 to 8.05
Attending	2.6	0.21 to 32.90	3.00	0.22 to 40.53
Propensity score	-	-	0.09	0.02 to 0.44

^a^Adjusted for all other variables shown; Hosmer-Lemeshow goodness-of-fit *p* value = 0.87; ^b^the composite complication includes hypotension, desaturation, or aspiration during intubation.

CI, confidence interval; PGY, postgraduate year, NIPPV, noninvasive positive-pressure ventilation; ARDS, acute respiratory distress syndrome.

## Discussion

These results demonstrate an increased risk of a composite complication associated with the intubation of critically ill patients who have failed NIPPV compared to patients intubated primarily without a trial of NIPPV. After controlling for potential confounding variables including a diagnosis of pneumonia or ARDS and factors known to increase complications of intubation, such as the number of attempts, operator experience, and the likelihood of being placed on NIPPV, there is an increased odds of a composite complication (hypotension, desaturation, or aspiration) when intubation is delayed due to a trial of NIPPV. When one of these complications occurs, these data show an association with increased unadjusted odds of death in the ICU. This is the first such association described in the literature and suggests a possible mechanism for the cautions raised by previous authors for the use of NIPPV in mixed respiratory failure [2,14,15,17,22,23,33].

Schnell and colleagues recently reported an increase in NIPPV use over a 15-year period for patients with respiratory failure requiring ventilator support and found that while NIPPV use increased over that time period (42% by 2011), NIPPV failure was an independent risk factor for mortality [2]. Only patients with acute on chronic respiratory failure showed a 60-day mortality benefit from the use of NIPPV, which has been demonstrated in other studies as well [7,21]. Most notably, Schnell et al. found a trend toward increased mortality in immunocompetent patients with respiratory failure, especially in hypoxemic respiratory failure, which has a high rate of intubation and a higher mortality when intubation is delayed by a trial of NIPPV [2,23].

Patients with acute hypoxemic respiratory failure treated with NIPPV require intubation in 30 to 84% [2,15,22,23,33] and have shown a higher mortality in some studies [15,17]. Gristina et al. found that NIPPV success decreased mortality (36%) in hematologic malignancy patients with acute lung injury compared to primary intubation (50%) [20]. However, intubation after failed NIPPV portended a 74% mortality rate. Although NIPPV has been successful in reducing intubation rates and mortality in patients with COPD exacerbations, NIPPV failure requiring intubation in this patient population has recently been shown to be associated with a 61% higher odds of mortality than COPD patients intubated primarily [1]. An Agency for Healthcare Research and Quality (AHRQ) report suggests that NIPPV should be used very cautiously, if at all, for respiratory failure not caused by COPD or CHF [14]. The question remains regarding the mechanism mediating the increased risk of death associated with NIPPV failure. Our study suggests that an increase in intubation complications may at least partially explain the increased mortality.

Reintubation for respiratory failure after failed extubation has been associated with higher complications and mortality than patients with a successful extubation [14,34-39]. Additionally, mortality worsens with longer delays in reintubation [34,38,40-42]. While NIPPV can reduce the reintubation rate in this population, failing NIPPV worsens prognosis, significantly delays reintubation and worsens mortality compared to earlier reintubation [18]. Esteban et al. evaluated NIPPV after extubation and found that patients that improved with NIPPV typically improved by 2 h, while patients that required reintubation were intubated at 2 h and 30 min [13,18].

Regardless of whether NIPPV is used or not, the intubation of critically ill patients poses an increased risk due to the complex anatomy, physiology, and limited reserve of these patients. Consequently, higher complication rates occur in these patients, including significant hypoxemia in up to 44% and cardiac arrest in 1 in 50 [28,43-48]. There is significant interest in methods of reducing these risks such as prolonging safe apnea time and preventing desaturation during intubation, including using NIPPV for alveolar recruitment in hypoxemic respiratory failure [49-51]. Positive end-expiratory pressure (PEEP) with the use of NIPPV can be useful for pre-oxygenating patients with shunt physiology prior to intubation [49]; however, our results show that delaying the intubation by using NIPPV may increase the intubation risk. Although NIPPV may improve pre-oxygenation, Delclaux reported a concerning number of patients with cardiac arrest upon intubation [16].

There are several important limitations to this study. The most important is the retrospective nature of the data collection. We attempted to mitigate this effect by adjusting for the likelihood of being placed on NIPPV and controlling for potential confounding variables that would increase the odds of a complication during intubation. While we attempted to adjust for the likelihood of being placed on NIPPV, only a randomized controlled trial will be able to answer this question definitively. For example, failed NIPPV can occur from poor patient selection with a high severity of illness or worsening of the disease with time while on NIPPV. We attempted to control for poor patient selection by including severity of illness in the propensity score; however, this may not completely account for the bias. The propensity score for receiving NIPPV was generated from variables expected to affect the decision to use NIPPV. There may be variables that might have contributed to the decision to use NIPPV that were not captured, such as the treating physician’s impression of the etiology of respiratory failure or the potential for difficulty with intubation not explained by the recorded DACs.

Selection bias may account for a difference in outcomes, particularly if some patients were selected for a trial of NIPPV because of predicted difficulty with intubation at the outset. Additionally, we examined all patient deaths in the ICU in the outcome. We feel that this is a more accurate representation of the risk of death, rather than excluding the patients where care was withdrawn given the variability in the decision to withdraw care and its expected dependence on clinical course. By nature of the dataset being patients that were intubated either primarily or after failed NIPPV, patients with a ‘do-not-intubate’ order related to their goals of care were naturally excluded. Lastly, the patient population these data were selected from were all patients intubated in the ICU, not all patients placed on NIPPV. Therefore, the success rate of NIPPV in our setting is not available. This is likely the explanation for the high percentage of NIPPV failure intubations due to hypoxemic respiratory failure. While these data show the presence of an association, they should be viewed as hypothesis generating and highlight the need for a randomized control trial to definitively examine the relationship between NIPPV failure, intubation complications, and mortality.

## Conclusions

These data show that intubation following failed NIPPV for acute respiratory failure is associated with increased odds of a composite complication (desaturation, hypotension, or aspiration) compared to patients intubated without a trial of NIPPV. Our findings highlight the pressing need for a rigorous investigation into the optimal use of NIPPV. Until data from prospective trials are available, early intubation and mechanical ventilation should be considered in any etiology of acute respiratory failure with a high rate of NIPPV failure, such as pneumonia, ARDS, or extubation failure.

## Abbreviations

ACGME: Accreditation Council for Graduate Medical Education
APACHE: acute physiology and chronic health evaluation
ARDS: acute respiratory distress syndrome
CCM: critical care medicine
CHF: congestive heart failure
COPD: chronic obstructive pulmonary disease
CQI: continuous quality improvement
DACs: difficult airway characteristics
GVL: GlideScope video laryngoscopy
ICU: intensive care unit
NIPPV: noninvasive positive-pressure ventilation
OTI: orotracheal intubation without medication
PGY: postgraduate year
Pulm/CCM: pulmonary and critical care medicine
RSI: rapid sequence intubation
SAPS: simplified acute physiology score
SED: sedation-only intubation
